# Targeting DYRK1A/B kinases to modulate p21‐cyclin D1‐p27 signalling and induce anti‐tumour activity in a model of human glioblastoma

**DOI:** 10.1111/jcmm.17002

**Published:** 2021-10-28

**Authors:** Andrew J. Massey, Karen Benwell, Mike Burbridge, Andras Kotschy, David Lee Walmsley

**Affiliations:** ^1^ Vernalis (R&D) Ltd Cambridge UK; ^2^ Institut de Recherches Servier Croissy‐sur‐Seine France; ^3^ Servier Research Institute of Medicinal Chemistry Budapest Hungary; ^4^ Present address: Engitix London UK

**Keywords:** DYRK1A, DYRK1B, glioblastoma, kinase inhibitor

## Abstract

The dual‐specificity tyrosine‐regulated kinases DYRK1A and DYRK1B play a key role in controlling the quiescence‐proliferation switch in cancer cells. Serum reduction of U87MG 2D cultures or multi‐cellular tumour spheroids induced a quiescent like state characterized by increased DYRK1B and p27, and decreased pRb and cyclin D1. VER‐239353 is a potent, selective inhibitor of the DYRK1A and DYRK1B kinases identified through fragment and structure‐guided drug discovery. Inhibition of DYRK1A/B by VER‐239353 in quiescent U87MG cells increased pRb, DYRK1B and cyclin D1 but also increased the cell cycle inhibitors p21 and p27. This resulted in exit from G0 but subsequent arrest in G1. DYRK1A/B inhibition reduced the proliferation of U87MG cells in 2D and 3D culture with greater effects observed under reduced serum conditions. Paradoxically, the induced re‐expression of cell cycle proteins by DYRK1A/B inhibition further inhibited cell proliferation. Cell growth arrest induced in quiescent cells by DYRK1A/B inhibition was reversible through the addition of growth‐promoting factors. DYRK inhibition‐induced DNA damage and synergized with a CHK1 inhibitor in the U87MG spheroids. *In vivo*, DYRK1A/B inhibition‐induced tumour stasis in a U87MG tumour xenograft model. These results suggest that further evaluation of VER‐239353 as a treatment for glioblastoma is therefore warranted.

## 1 INTRODUCTION

The CMGC family DYRK (dual‐specificity tyrosine‐regulated) kinases consist of two classes: Class 1 (DYRK1A and DYRK1B) and Class 2 (DYRK2, DYRK3 and DYRK4) both of which are capable of phosphorylating serine‐threonine and tyrosine residues.^1^, ^2^ The Class 1 DYRKs are potentially interesting drug targets as they have been implicated in a range of human conditions including Down Syndrome,^3^ neurodegenerative disorders such as Alzheimer's^4^ and Parkinson's disease,^5^ and cancer.^6^, ^7^, ^8^

DYRK1A and 1B play an important role in cell cycle control. These kinases regulate the induction of quiescence via control of the DREAM complex^9^ via binding to and the subsequent phosphorylation of serine 28 of LIN52 thereby controlling the G0‐G1 transition and exit from quiescence.^1^, ^10^, ^11^ Additionally, DYRK1A and 1B can phosphorylate cyclin D1 (resulting in its destabilization)^12^, ^13^, ^14^ and p27Kip1 (increasing its stabilization)^15^, ^16^, ^17^ thereby controlling the S‐phase checkpoint. DYRK1B expression is increased in quiescent cells and inhibition of DYRK1B can sensitize quiescent pancreatic and ovarian carcinoma cell lines to DNA damaging agents^18^, ^19^ and quiescent GIST cells to imatinib.^11^

We have previously described the use of fragment and structure‐based lead discovery methods to identify potent and selective ATP competitive inhibitors of DYRK1A and DYRK1B.^20^ The structure and key data of VER‐239353 (compound **34** in^20^) are reproduced in Figure S1. Here, we further evaluate the anti‐tumour activity of this compound in a model of human glioblastoma.

## 2 MATERIALS AND METHODS

### 2.1 Materials

VER‐239353 was from Vernalis (R&D) Ltd and prepared as a 20 mM DMSO stock, aliquoted and frozen at −20°C. The same stock was used for all experiments.

### 2.2 Cell lines and cell culture

All cell lines were purchased from the American Type Culture Collection (ATCC), established as a low passage cell bank and then routinely passaged in our laboratory for less than 3 months after resuscitation. A375, PANC‐1, PC‐3 and U87MG cells were routinely cultured in DMEM or NCI‐H1299 in RPMI‐1640 containing 10% foetal calf serum (FCS) and 1% penicillin / streptomycin at 37°C in a normal humidified atmosphere supplemented with 5% CO_2_. Cells were authenticated by STR profiling (LGC Standards, Teddington UK).

For quiescence induction, cells were trypsinized and resuspended in media with 10% FCS, centrifuged and washed twice with FCS‐free media and then resuspended in media containing 0.2% FCS and counted. Cells were subsequently plated in media containing 0.2% FCS and incubated for 72 h before analysis. Multi‐cellular tumour spheroids were generated as previously described.^21^

### 2.3 Growth inhibition assays

5000 cells per well were seeded in 96‐well plates and incubated overnight. Cells were treated with a 10‐point titration of compound for 72 h. The effect on cell proliferation was determined using Cell Titer Glo reagent (Promega).

### 2.4 In vitro combination synergy

Pre‐formed U87MG spheroids were treated with a combination of VER‐239353 and the CHK1 inhibitor VER‐157932^22^ for 7 days at fixed ratio concentrations. Synergy was calculated using the Median Dose Effect in CalcuSyn software (Biosoft, UK) according to the method of Chou and Talalay.^23^

### 2.5 High content cell cycle analysis

Determination of cell cycle fractions was conducted using high content imaging as previously described.^24^ For multiparametric cell cycle analysis, cells were labelled with 10 μM EdU for 15 min immediately prior to fixation with 3.7% paraformaldehyde in PBS at room temperature for 15 min. Cells were washed twice in PBS then twice in 3% BSA in PBS before permeabilization with 0.5% Triton X100 in PBS for 20 min at room temperature. Cells were washed twice with 3% BSA in PBS before incorporated EdU was labelled with an Alexa Click‐iT EdU labelling kit (Life Technologies). Following blocking for 30 min with 5% normal goat serum in PBS, cells were incubated with an anti‐pHH3 (S10) primary antibody diluted in antibody dilution buffer (1% BSA, 0.3% Triton X100 in PBS) at 4°C for 16 h. Cells were washed with PBS then incubated with an Alexa‐labelled secondary antibody (1:500, Life Technologies) and Hoechst 33342 (1 μg/ml) in antibody dilution buffer at room temperature for 60 min. Following washing with PBS, cells were imaged with an Operetta high content imaging system (Perkin Elmer) at 10× magnification and analysed using Harmony software (Perkin Elmer). The antibodies used are listed in Table S1.

### 2.6 Immunoblotting

Cells were washed once with PBS and lysed in RIPA buffer containing protease and phosphatase inhibitor cocktail (Roche). Protein concentration was determined using a BCA kit (Pierce). Equal amounts of lysate were separated by SDS‐PAGE and Western blot analysis conducted using the antibodies indicated in Table S1. Primary antibodies were detected with HRP‐conjugated secondary antibody (Santa Cruz Biotechnology) and detected with Western Lightning (Perkin Elmer) or Immobilon (Millipore) chemiluminescent HRP substrate. Blots were imaged using an LAS 4000 luminescence imager (Fujifilm). Densitometry was determined using Image J software (NIH).

### 2.7 High content immunofluorescent imaging

Following compound treatment, cells were fixed in 3.7% paraformaldehyde in PBS at room temperature for 15 min, washed with PBS, blocked with 5% normal goat serum in 0.3% Triton X100 in PBS for 1 h at room temperature then incubated with primary antibody diluted in antibody dilution buffer (1% BSA, 0.3% Triton X100 in PBS) at 4°C for 16 h. Cells were washed with PBS then incubated with an Alexa‐labelled secondary antibody (1:500, Life Technologies) and Hoechst 33342 (1 μg/ml) in antibody dilution buffer at room temperature for 60 min. Following washing with PBS, cells were imaged with an Operetta high content imaging system (Perkin Elmer) at 10× or 20× magnification and analysed using Harmony software (Perkin Elmer).

### 2.8 In vivo efficacy in U87MG xenografts

Female nude mice (Crl:NU(NCr)‐Foxn1nu) were purchased from Charles River Laboratories. Xenograft studies were undertaken by Charles River Laboratories Discovery Services, North Carolina and accredited by the Association for Assessment and Accreditation of Laboratory Animal Care International. The U87MG tumour line was maintained by serial SC transplantation in female athymic nude mice. Tumour fragments, approximately 1 mm^3^ each, were implanted SC into the right flank of each animal and allowed to grow towards a target size of 100–150 mm^3^. On day 1 of the study, tumours were randomized into treatment groups before compound administration.

VER‐00239353 was formulated in 60 mM HCl and 40% hydroxypropyl‐β‐cyclodextrin and administered by oral gavage once daily for 21 days. Tumour size was measured twice weekly with electronic callipers and tumour volume calculated according to the formula ((width × width) × length)/2. Body weight was measured daily for the first 5 days then twice weekly thereafter. The study endpoint was defined as a tumour volume of 2000 mm^3^ or D28, whichever came first. Each animal was euthanized when its tumour reached the endpoint.

TGI (%) = 1 − ((T_11_/T_0_)/(C_11_/C_0_))/1 − (C_0_/C_11_) × 100 where T_11_ and T_0_ equals mean tumour volume of treated group at day 11 and day 1, respectively, and C_11_ and C_0_ equals median tumour volume of control group at day 11 and day 1, respectively. Significance was determined using a one‐way ANOVA in GraphPad PRISM 8.4.3.

The time to endpoint (TTE) for each mouse was calculated from the following equation: TTE = log10 (endpoint volume) – b/m where b is the intercept and m is the slope of the line obtained by linear regression of a log‐transformed tumour growth data set. The data set is comprised of the first observation that exceeded the study endpoint volume and the three consecutive observations that immediately preceded the attainment of the endpoint volume. Animals that did not reach endpoint were euthanized at the end of the study and assigned a TTE value equal to the last day of the study (D28). Animals determined to have died from treatment‐related (TR) causes were assigned a TTE value equal to the day of death. If the animal died from non‐treatment‐related (NTR) causes, it was excluded from the TTE calculations.

## 3 RESULTS

### 3.1 DYRK1/A inhibition of serum‐starved cancer cells induces re‐expression of cell cycle proteins as well as cell cycle inhibitors

**FIGURE 1:**
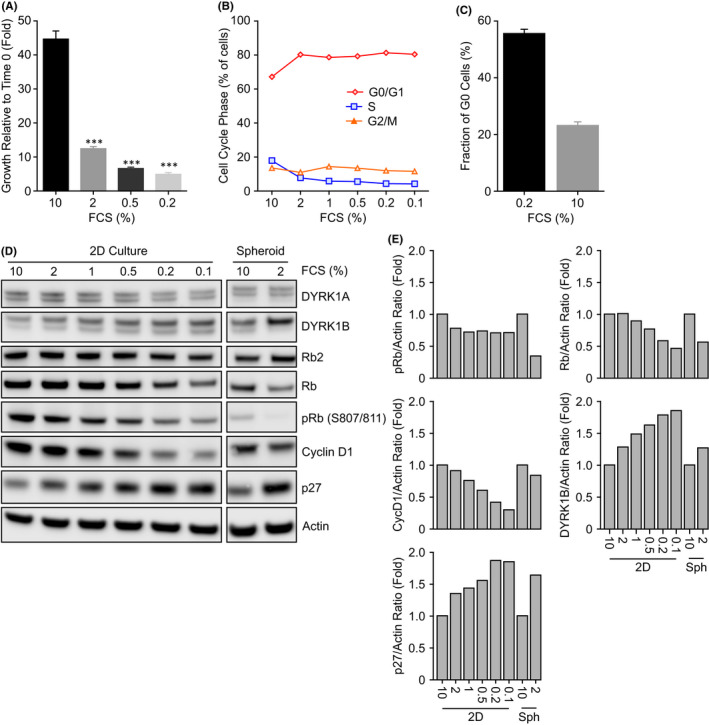
Reduction of serum induces a quiescent like state in U87MG glioblastoma cells. (A) U87MG cells were grown in media containing the indicated concentrations of FCS for 168 h. Cell proliferation was determined using Cell Titre Glo. Values are the mean of 8 determinations ± SD. (B) Cell cycle profiles were determined on U87MG cells grown in media containing 0.1 to 10% FCS for 72 h. Values are from a single determination. (C) U87MG cells were grown as in B then fixed, stained for Ki67 expression and analysed by high content immunofluorescence. G0 cells were Ki67 negative with a G1 DNA content. Mean ± SD, *n* = 8 independent wells. (D) Protein biomarker changes in U87MG cells cultured in 0.1 to 10% FCS in or as multi‐cellular tumour spheroids for 72 h were determined by immunoblotting. The experiment was conducted twice and an example blot is shown. (E) The data in (D) was quantified and the ratio to Actin determined

The 2D culture of U87MG cells in low serum (0.5% FCS or less) resulted in reduced cell proliferation (Figure 1A), a reduction in cycling cells (Figure 1B) and an increase in the fraction of cells staining negative for Ki67 (indicative of G0 phase) from 23.0 to 55.4% (Figure 1C). This increase in quiescence was also evident following Western blot analysis of U87MG cells grown in reduced serum. A reduction in phosphorylation of Rb at S807/811, a decrease in total Rb and cyclin D1 protein, and an increase in DYRK1B and p27 expression were observed (Figure 1D). Likewise, formation and growth of U87MG multi‐cellular tumour spheroids in lower (2% compared to 10%) FCS resulted in a similar decrease in Rb S807/811 phosphorylation, total Rb and cyclin D1 protein and an increase in DYRK1B and p27 expression (Figure 1D).

**FIGURE 2:**
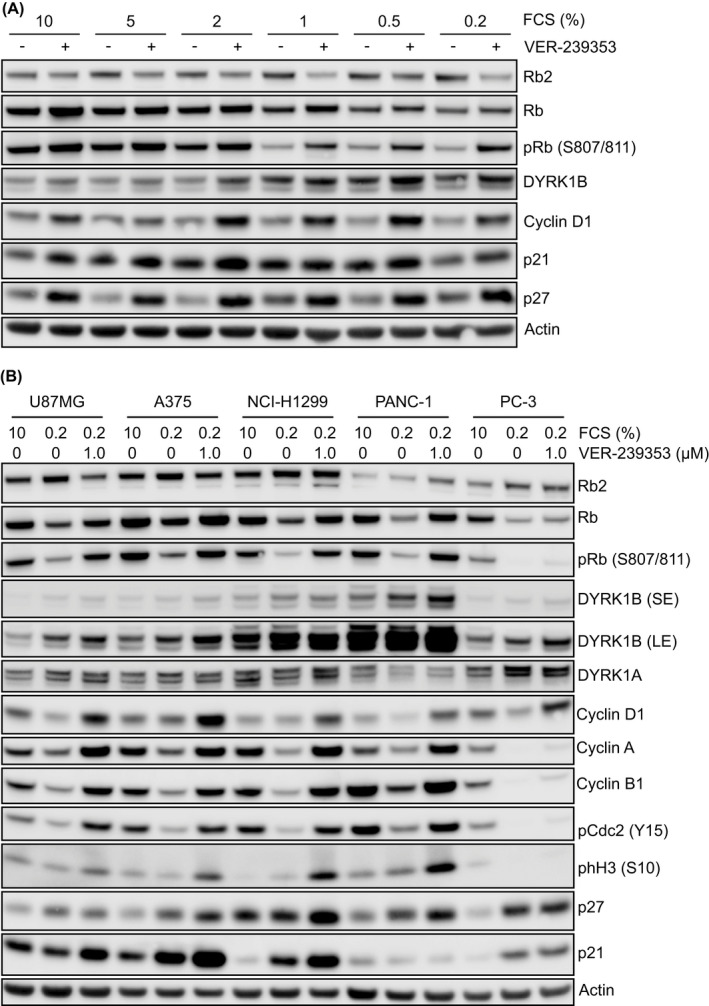
Upregulation of p21, p27 or cell cycle protein expression in quiescent cancer cells following DYRK1A/B inhibition was cell line dependent. (A) U87MG cells were cultured in 10 to 0.2% FCS for 72 h before being treated with 0 (−) or 1 μM (+) VER‐239353 for 24 h. (B) p53 wild‐type (U87MG, A375 or NCI‐H1299) or p53 mutant (PANC‐1 or PC‐3) cells were grown in 0.2 or 10% FCS for 72 h before treatment with 0 or 1 μM VER‐239353 for a further 48 h. Protein marker changes were determined by immunoblotting. SE, short exposure; LE, long exposure

VER‐239353 is a potent selective inhibitor of DYRK1A/1B with *in vitro* IC_50_s of 7 and 2.4 nM, respectively, and >30‐fold selectivity versus DYRK2. Inhibition of DYRK1A/B by VER‐239353 induced the phosphorylation of Rb and increased the expression of DYRK1B and cyclin D1 in U87MG cells grown in reduced serum (Figure 2A and Figure S2A). p27 and p21 expression were also increased but this occurred independently of serum concentration. This observation was not limited to U87MG cells. Growth of A375, NCI‐H1299, PANC‐1 or PC‐3 cells in low serum (0.2% FCS) resulted in reduced Rb phosphorylation and a decrease in levels of cyclin D1, A and B1 and pCdc2(Y15) (Figure 2B and Figure S2B). Inhibition of DYRK1A/B by VER‐239353 resulted in re‐phosphorylation of Rb and Cdc2(Y15) and increased expression of cyclin D1, A and B1, and p27.

The reactivation and re‐expression on cell cycle proteins in serum‐starved U87MG cells by VER‐239353 occurred in a dose‐dependent fashion (Figure S3A) with EC_50_ values for cyclin D1, p21 and DYRK1B of 118, 474 and 282 nM, respectively (Table S2). These changes happened rapidly within 24 h of VER‐239353 addition and were sustained for at least 96 h (Figure S3B,C). These changes to cell cycle protein phosphorylation and expression were not just restricted to U87MG cells grown as 2D monolayers. Treatment of U87MG cells grown as multi‐cellular spheroids in either 2 or 10% FCS with VER‐239353 resulted in a dose‐dependent increase in pRb and total DYRK1B, cyclin D1, p21 and p27 (Figure S4A,B). The effects were more marked in the spheroids growing in 2% FCS compared to those growing in 10% FCS with EC_50_ values for cyclin D1, p21 and DYRK1B of 132, 94 and 103 nM, respectively (Figure S4C and Table S2).

**FIGURE 3:**
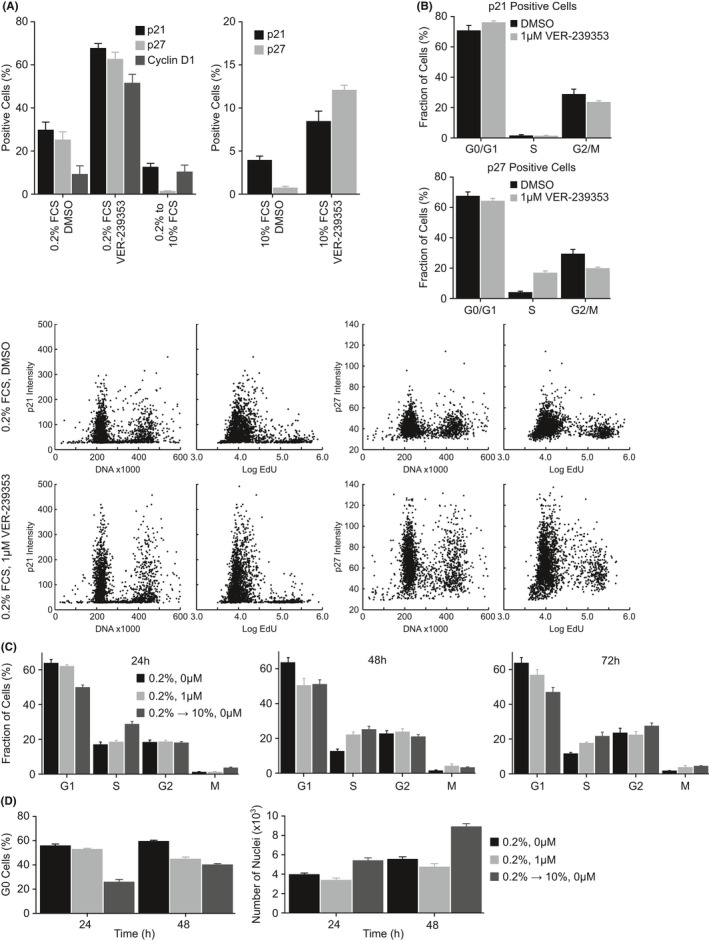
Inhibition of DYRK1A/B induces re‐expression of cell cycle proteins as well as cell cycle inhibitors in quiescent U87MG glioblastoma cells. (A) U87MG cells were cultured in 0.2% FCS for 72 h before being treated with 0 or 1 μM VER‐239353 for 24 h. p21, p27 or Cyclin D1 expression was determined by high content immunofluorescent imaging. Marker positive nuclei were quantified using an Operetta high content imaging system and Harmony software. Values are the mean of 8 determinations ± SD. (B) The cell cycle distribution of the p21 and p27 positive U87MG cells in 0.2% FCS treated with DMSO or 1 µM VER‐239353 were determined by high content imaging. (C) U87MG cells were cultured in 0.2% for 72 h then treated with either DMSO or 1 µM VER‐239353 (still in 0.2% FCS) or DMSO in 10% FCS. The fraction of cells in G1, S, G2 or M determined at the indicated time points using the Operetta high content instrument and Harmony software. Values are the mean of 8 determinations ± SD. (D) The G0 fraction of U87MG cells was determined by Ki67 staining

These observations were confirmed by high content immunofluorescent imaging. Treatment of U87MG cells grown in low FCS with VER‐239353 dramatically increased the percentage of cells staining positive for p21, p27 or cyclin D1 (Figure 3A). In comparison, switching the cells from growth in low (0.2%) to high (10%) serum did not. In cells growing in high serum, VER‐239353 treatment also increased the fraction of cells staining positive for p21 or p27 but the magnitude of effect was much lower than that in low serum. An analysis of the cell cycle phase (using DNA and EdU content) by high content imaging revealed that the p21 and p27 positive cells following growth in low serum were in the G0/G1 phase. VER‐239353 treatment did not significantly alter the cell cycle distribution of the p21 or p27 positive cells with the majority still in G0/G1 (Figure 3B). Switching U87MG cells grown in low serum (0.2%) to high serum (10%) resulted in an obvious re‐entry into S‐phase after 24 h (Figure 3C). In comparison, S‐phase re‐entry after inhibition of DYRK1A/B was much more delayed. An increase in S‐phase cells after DYRK1A/B inhibition was not observed until at least 48 h. This correlated with the decrease in the apparent fraction of G0 cells with a decreased G0 cells observed after 24 h in cells switched to high serum whilst effects in the DYRK1A/B inhibited cells was apparent after only 48 h (Figure 3D). This was also apparent in the cellular morphology, with U87MG cells grown in low serum and then treated with VER‐239353 retaining the morphology (reduced nuclear and cellular area, high cell roundness) of cells grown under the same conditions treated with DMSO (Figure S5). In comparison, cells switched from low to high serum had a greater cell area and reduced cell roundness compared to those grown in low serum.

### 3.2 DYRK1A/B inhibition inhibits the proliferation of quiescent U87MG cells and U87MG multi‐cellular tumour spheroids

**FIGURE 4:**
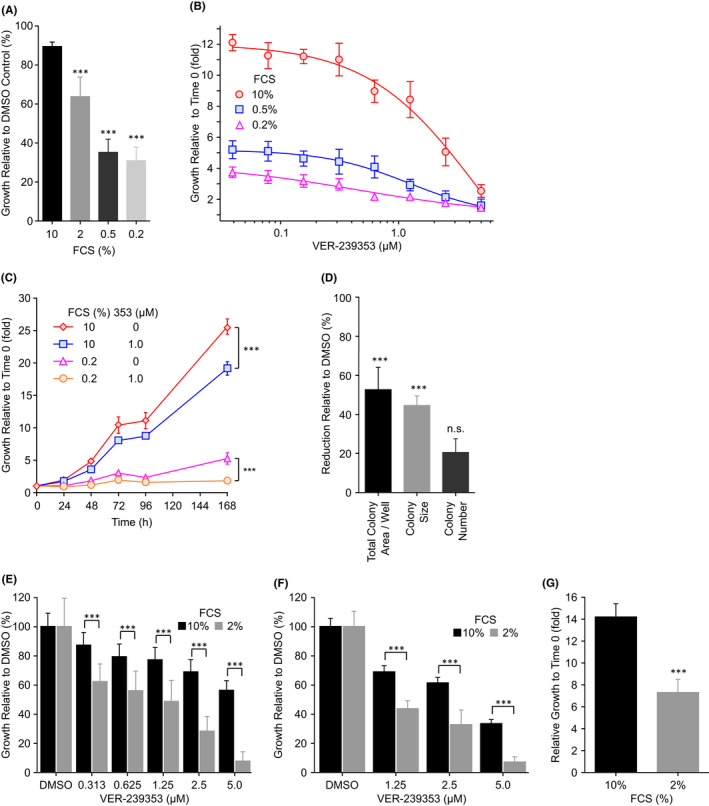
VER‐239353 inhibits the proliferation of quiescent U87MG cells and U87MG multi‐cellular tumour spheroids. (A) U87MG cells cultured in 0.2 to 10% FCS were treated with 1.25 μM VER‐239353 for 168 h. (B) U87MG cells cultured in 0.2, 0.5 or 10% FCS were treated with VER‐239353 for 168 h. Growth was calculated relative to Day 0. (C) U87MG cells cultured in 0.2 or 10% FCS were treated with 0 or 1 μM VER‐239353. Cell proliferation was determined at the indicated time points. (D) U87MG cells cultured in 0.2% FCS were treated with 1.25 μM VER‐239353 for 168 h. Colony area and number were determined using an Operetta high content instrument. (E) U87MG multi‐cellular tumour spheroids were formed in 2% or 10% FCS for 3 days before treatment with 0–5 μM VER‐239353 for a further 7 days (reproduced from Figure S4B in^20^). (F) U87MG multi‐cellular tumour spheroids were formed in the presence of 0–5 μM VER‐239353 for 7 days. (G) Comparison of the relative growth rates of U87MG multi‐cellular tumour spheroids in 2% or 10% FCS. Cell viability was determined using Cell Titer Glo. Values are the mean of 8 replicates ± SD

Inhibition of DYRK1A/B by VER‐239353 (^20^, Figure S1A,B) reduced the proliferation of U87MG cells with the effects on cell proliferation much more pronounced under low serum growth conditions (Figure 4A,B) and longer treatment duration (Figure 4C). VER‐239353 GI_50_ values were 4.6, 1.2 and 0.44 µM in U87MG cells grown in 10, 0.5 and 0.2% serum, respectively. In a colony formation assay, treatment of U87MG cells with 1.25 µM VER‐239353 had a small effect on the number of viable colonies reducing the number of viable colonies by around 20% but a more pronounced effect on the size of those viable colonies (Figure 4D). VER‐239353 inhibited the proliferation of U87MG cells grown as multi‐cellular tumour spheroids (Figure 4E,F) with activity again dependent on serum concentration. VER‐239353 was approximately 4.8‐fold more potent in the lower serum (2% FCS, GI_50_ 1.2 µM) compared to the higher (10% FCS, GI_50_ 5.8 µM) conditions. Under lower serum conditions, U87MG multi‐cellular tumour spheroids exhibited approximately half the proliferation rate of those grown in higher serum (Figure 4G). This decrease in U87MG proliferation by VER‐239353 did not appear due to decreased expression of EGFR (Figure S6A). A decrease in EGFR expression was observed only in U87MG cells treated with 10 μM VER‐239353 (61% reduction, Figure S6B). This was in direct contrast to results published previously with Harmine (Pozo et al., 2013).

### 3.3 Inhibition of DYRK1A/B does not prevent proliferation of U87MG cells when cell growth signals are restored

**FIGURE 5:**
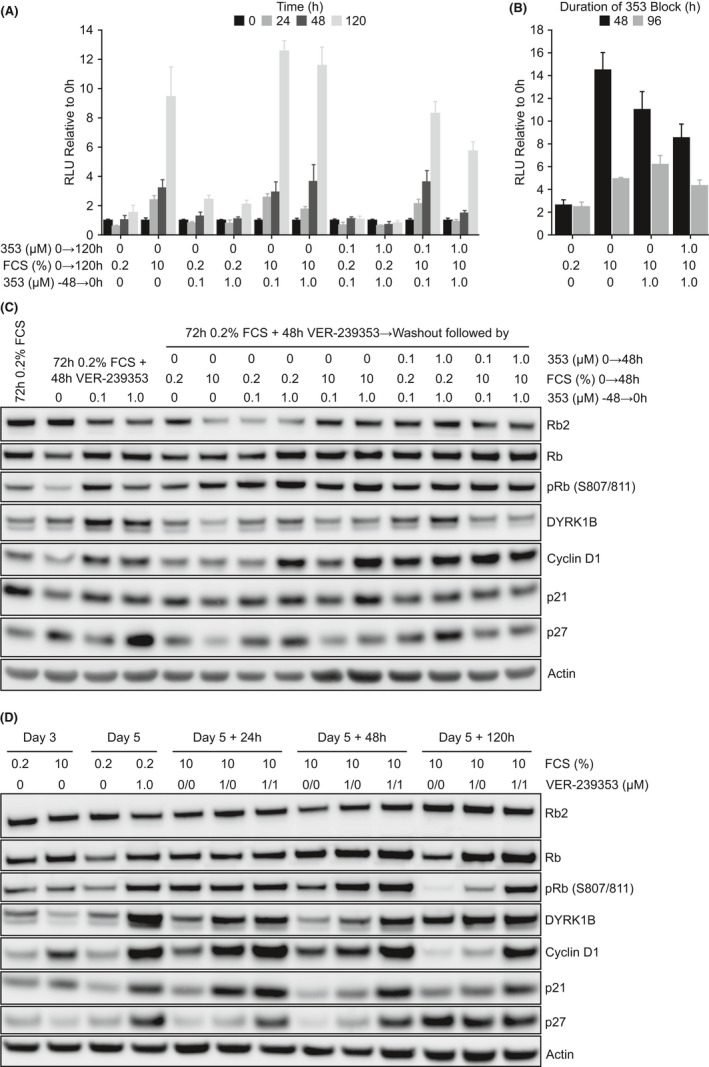
Inhibition of DYRK1A/B does not prevent U87MG cells proliferating when cell growth signals were restored. (A) U87MG cells were cultured in 0.2% FCS for 72 h before being treated with 0, 0.1 or 1.0 μM VER‐239353 for a further 48 h. Media was removed and replaced with fresh media containing 0.2 or 10% FCS and 0, 0.1 or 1.0 μM VER‐239353. Cell viability was determined using Cell Titer Glo 24, 48 or 120 h later. (B) U87MG cells were cultured in 0.2% FCS for 72 h before being treated with 0 or 1.0 μM VER‐239353 for a further 48 or 96 h. Media was removed and replaced with fresh media containing either 0.2% FCS and 0 μM VER‐239353 or 10% FCS and 0 or 1.0 μM VER‐239353. Cell viability was determined using Cell Titer Glo 120 h later. Values are the mean of 8 determinations ± SD. (C) U87MG cells were treated as in (A), and protein changes determined by immunoblotting 48 h after the washout. (D) U87MG cells were cultured in 0.2% FCS for 72 h before being treated with 0 or 1.0 μM VER‐239353 for a further 48 h. Media was removed and replaced with fresh media containing 10% FCS and 0 or 1.0 μM VER‐239353. Protein lysates were prepared 24, 48 or 120 h after this media change and protein changes determined by immunoblotting

We have demonstrated that DYR1A/B inhibition with VER‐239353 in U87MG cells growing in low serum induces the reactivation or re‐expression of cell cycle driving genes (such as pRb and cyclin D1) but also of the cell cycle inhibitors p21 and p27. Subsequently, we evaluated whether DYRK1A/B inhibition could prevent these cells from proliferating when cell growth signals (ie, high serum) were restored. U87MG cells were cultured in low serum for 72 h, treated with VER‐239353 for 48 h (still in low serum), then released into high serum in the absence or presence of VER‐239353. In all cases, release into high serum induced the U87MG cells to proliferate (Figure 5A) with cells released into high serum media without VER‐239353 able to proliferate more rapidly than those where VER‐239353 remained. Increasing the duration of VER‐239353 block prior to release into high serum from 48 to 96 h did not alter the results. A longer duration of growth in low serum reduced the ability of cells to reproliferate in high serum independent of VER‐239353 (Figure 5B). Analysis of cell cycle proteins revealed that high serum led to increased Rb phosphorylation and cyclin D1 expression in cells pre‐treated with VER‐239353. The expression levels of p21 and p27 were reduced compared to cells grown in low serum plus VER‐239353 but were still higher than that observed in control cells grown only in high serum (Figure 5C,D and Figure S7A,B).

### 3.4 DYRK1A/B inhibition induces DNA damage in U87MG multi‐cellular spheroids and synergizes with CHK1 inhibition

**FIGURE 6:**
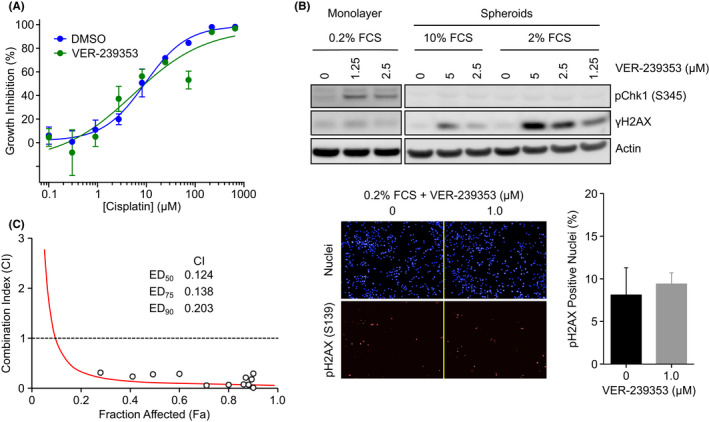
DYRK1A/B inhibition induces DNA damage in U87MG multi‐cellular spheroids and synergizes with CHK1 inhibition. (A) U87MG spheroids in 2% FCS were treated with a combination of increasing concentrations of cisplatin plus DMSO or 2.5 μM VER‐239353 for 7 days. Mean of 3 independent wells ± SD. (B) Monolayer U87MG cells were cultured in cultured in 0.2% FCS for 72 h then treated with VER‐239353 for a further 48 h. U87MG multi‐cellular tumour spheroids were formed in 2% or 10% FCS for 72 h before being treated with VER‐239353 for a further 72 h. Protein changes were determined by immunoblotting. The number of pH2AX (S139) positive nuclei was determined using an Operetta high content imager and quantified with Harmony software. All values are the mean of 8 determinations ± SD. (C) Combination index (CI) plots of VER‐239353 with CHK1 inhibitor concomitant treatment in U87MG spheroids. Values are the mean of 2 independent experiments

Previous studies have demonstrated that DYRK1A/B inhibition can increase the sensitivity of quiescent cancer cells to genotoxic drugs such as cisplatin and gemcitabine.^18^, ^25^, ^26^ U87MG tumour spheroids grown in low serum exhibited approximately equal sensitivity to cisplatin when treated in combination with VER‐239353 (GI_50_ 6.5 μM) compared to combination with DMSO (GI_50_ 9.4 μM) (Figure 6A). Treatment of U87MG multi‐cellular spheroids with VER‐239353 resulted in increased DNA damage as measured by an increase in expression of γH2AX (Figure 6B). This was more apparent when the cells were grown in lower (2%) serum compared to the standard 10% FCS and appeared to be independent of the ATR DNA damage response pathway as no increase in serine 345‐phosphorylation of CHK1 was observed. No increase in γH2AX was observed when the U87MG cells were grown as a 2D monolayer in low (0.2%) serum. Subsequent synergy between DYRK1A/B and CHK1 inhibition was observed in the U87MG spheroids grown in low serum with combination index (CI) values <1.0 at the ED_50_, ED_75_ and ED_90_ (Figure 6C).

### 3.5 VER‐239353 inhibits the growth of U87MG tumour xenografts

**FIGURE 7:**
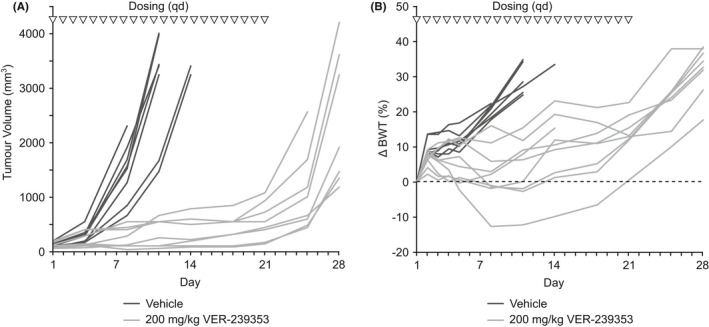
VER‐239353 inhibits the growth of U87MG glioblastoma xenografts. Nude mice with established U87MG subcutaneous tumour xenografts were dosed orally with 200 mg/kg VER‐00239353 daily for 21 days (triangles). Tumour volume (A) was determined by twice weekly calliper measurement and body weight (B) recorded. Values for each individual animal (*n* = 8 per group) are shown

U87MG tumours grafted subcutaneously in nude mice grow rapidly reaching the endpoint of 2000 mm^3^ in a median time of 9.1 days. VER‐239353 administered orally to nude mice bearing established U87MG glioblastoma tumours resulted in significant inhibition of tumour growth. Administration of 200 mg/kg VER‐239353 daily for 21 days induced virtual tumour stasis (Figure 7A, TGI = 91% at day 11, *p* < 0.001) with the effects on tumour growth maintained for the duration of dosing. However, on cessation of VER‐239353 dosing, tumours regrew rapidly with a growth rate similar to that of the vehicle‐treated tumours. This resulted in a median TTE of 27.5 days and TGD of 222% (*p* < 0.001). VER‐239353 was generally well tolerated when dosed as a single daily oral dose with no treatment‐related deaths observed. There were noticeable differences in body weight changes following VER‐239353 dosing across the cohort of 8 treated mice with one mouse demonstrating a 12–13% loss in body weight between days 8 and 14 whilst others demonstrated up to a 23% increase in body weight over the same period (Figure 7B). Any body weight loss was reversible with mice rapidly recovering body weight upon cessation of dosing. There was no apparent correlation between body weight changes and tumour growth inhibition.

## 4 DISCUSSION

VER‐239353 is a potent, selective inhibitor of DYRK1A and 1B kinases that exhibited significant anti‐tumour activity in the U87MG model of human glioblastoma both *in vitro* and *in vivo*. *In vitro*, U87MG cells exhibited much greater sensitivity when the cells were grown under lower serum conditions. This was even more apparent when they were grown anchorage‐independently as multi‐cellular tumour spheroids, a condition that is more representative of the *in vivo* situation. In a mouse U87MG tumour xenograft model, VER‐239353 exhibited significant anti‐tumour activity with almost complete tumour stasis.

DYRK inhibitors have previously demonstrated activity against human glioblastoma models through EGFR destabilization.^27^, ^28^ This is reported to occur through the activation of p53‐MDM2.^29^ The reason for the lack of EGFR downregulation at target‐hitting concentrations in our studies is not clear, although the discrepancy may be related to the fact that these published studies used either shRNA knockdown of DYRK1A, rather than inhibition of its kinase activity, or the less‐selective DYRK1A inhibitor Harmine. Selective inhibitors of CLK1/4 have been demonstrated to decrease EGFR.^30^ Most inhibitors of DYRK1A/1B also inhibit the CLK family of splicing kinases (at least at the level of *in vitro* kinase assays) and this difference may reflect differences in activity of these inhibitors on CLKs at the cellular level. Alternatively, the conditions under which the effect of small molecule DYRK inhibitors on EGFR levels was assayed may account for the differences. In Pozo et al,^27^ the effects of Harmine on EGFR levels were determined in serum‐starved cells stimulated with EGF. They suggest that DYRK1A controls the rate at which EGFR was degraded with DYRK1A inhibition increasing the rate of EGFR degradation and this occurred without affecting mRNA levels. DYRK1A has been demonstrated to interact with the E3 ligase TRAF2 leading to K63‐mediated ubiquitination of DYRK1A. This ubiquitination mediates DYRK1A translocation to vesicle membranes where it can phosphorylate Sprouty 2 resulting in inhibition of EGFR degradation.^31^ Silencing of DYRK1A or TRAF2 increases EGFR degradation and inhibition of the growth of glioma cells.

DYRK1A and DYRK1B have been demonstrated to play a role controlling the entry into senescence in a range of human cancer types including pancreatic,^32^, ^33^, ^34^ colon,^10^ ovarian^35^, ^36^ and squamous cell^37^ carcinomas and GIST.^11^ The mechanism(s) for this appear to be via the regulation of cyclin D1 and p27 stability, as well as control of the DREAM complex via LIN52. Inhibition of DYRK1A/B drives tumour cells out of quiescence (G0) and back into the cell cycle. Inhibitors therefore have the potential to target quiescent, chemo‐resistant cell populations.^1^ As such, inhibitors of DYRK1A/B have been shown to sensitize quiescent cancer cells to imatinib^11^ and DNA damaging agents such as cisplatin^18^, ^25^, ^26^ and gemcitabine^18^ through reversal of quiescence and increased cell cycling.

In the study presented here, we did not observe increased cell cycling of quiescent glioblastoma cells upon DYRK1A/B inhibition with VER‐239353. Instead, we observed a transition of cells from G0 to G1 which subsequently did not enter into S‐phase and cycling but remained arrested in G1. Increases in cyclin D1 and pRb were observed, two factors involved in initiating the cell cycle, but this was counterbalanced by an increase in the cell cycle inhibitors p21 and p27. A recent paper by Recasens and colleagues^38^ suggests that DYRK1A can induce cyclin B1 degradation and thereby control the activity of CDK1. Complete DYRK1A inhibition, as would be expected here with VER‐239353, resulted in cyclin B1 stabilization and accumulation in glioblastoma cells leading to CDK1 inhibition and cell cycle arrest. Here, we also observed increased cyclin B1, A and D1 expression in U87MG glioblastoma cells following complete DYRK1A/B inhibition with VER‐239353. In addition to inhibiting CDK1, this increased expression of cyclins could also lead to CDK2 inhibition further compounding the cell cycle arrest effects observed due to p21 and p27 accumulation.

In Down syndrome, increased DYRK1A expression increases G1 cell cycle duration through phosphorylation and degradation of cyclin D1. Subsequent DYRK1 inhibition increases cyclin D1 resulting in two cell subpopulations—one with accelerating cell proliferation and the other arresting through co‐stabilization of cyclin D1 and p21.^14^ Therefore, in this glioblastoma model, DYRK1 inhibition may favour the arrested subpopulation with high cyclin D1 but also high p21 levels over a population with increased cell proliferation. DYRK1A/B phosphorylates p27 on serine 10 leading to its stabilization^15^, ^17^ with depletion of DYRK1A/B resulting in decreased phosphorylation and destabilization.^15^, ^17^ However, in U87MG cells treated with VER‐239353, we observed the opposite. DYRK1A/B inhibition led to increased, not decreased, p27 expression. Evidence exists that this site can be phosphorylated by other kinases with these kinases therefore potentially able to increase p27 independently of DYRK1A/B, and when DYRK1A/B is inhibited. This increased expression of p21, p27 and cyclin D1 following DYRK1A/B inhibition might be due to altered protein stability following loss of phosphorylation but may equally be due to changes at the mRNA transcript level. Further work is needed to understand this.

Inhibiting DYRK1A/B in the glioblastoma model with VER‐239353 forced the cells to exit G0 and enter G1 but not actively undergo cell cycling. Therefore, given this lack of cell cycling, these quiescent U87MG cells would not be predicted to be sensitized to cytotoxic chemotherapy by DYRK1A/B inhibitors (because such drugs require cell cycle progression to be therapeutic). This was the case observed where U87MG spheroids grown in low serum exhibited an equal sensitivity to cisplatin in the absence or presence of VER‐239353. However, in the clinic, driving cells out of the quiescent niche through DYRK1A/B inhibition and the subsequent reactivation of cell cycling by other factors (eg, mitogens) may result in increased sensitivity to cytotoxic chemotherapy. Additionally, DYRK1A/B inhibition may sensitize glioblastoma cells to drugs that exert their effects in the G1 phase of the cell cycle, for example CDK4 or MEK inhibitors. Such drugs tend to induce G1/S arrest so may only be additive with DYRK1A/B inhibitors.

We observed increased DNA damage in U87MG multi‐cellular spheroids treated with the DYRK1A/B inhibitor and synergy when combined with an inhibitor CHK1. CHK1 inhibitors are currently in Phase 1 and 2 trials.^39^ Several strands of evidence suggest a link between DNA damage response and repair pathways and the DYRK1A and B kinases. The E3 ligase RNF169 has been demonstrated to be a substrate of DYRK1A with one phosphorylation site suggested to be critical for the ability of RNF169 to displace 53BP1 from sites of DNA damage.^40^ DYRK1A depletion in this study sensitized cells to ionizing radiation. The interaction of DYRK1A with RNF169 was confirmed in a second study.^41^ However, in this study, U2OS cells devoid of DYRK1A had increased homologous recombination repair efficiency and increased resistance to DNA damage.

Given CHK1's role as a central component of the DNA damage response, further evaluation of DYRK1A/B inhibitors with CHK1 inhibitors as a potential therapeutic combination would be interesting.

In summary, these results demonstrate the potential of DYRK1A/B inhibition to push tumour cells back into the cell cycle and hence re‐sensitize to chemotherapy or targeted therapies. Combined with the marked *in vivo* activity of VER‐239353 in the U87MG glioblastoma model, this suggests that VER‐239353 is worthy of further evaluation as a treatment for this hard‐to‐treat disease.

## CONFLICT OF INTEREST

All authors are employees of either Vernalis (R&D) Ltd or Servier. This work was supported by Servier and Vernalis (R&D) Ltd.

## AUTHOR CONTRIBUTIONS

**Andrew J. Massey:** Conceptualization (lead); investigation (lead); methodology (lead); supervision (equal); writing–original draft (lead). **Karen Benwell:** Conceptualization (equal); supervision (equal); writing–review and editing (equal). **Mike Burbridge:** Conceptualization (equal); supervision (equal); writing–review and editing (equal). **Andras Kotschy:** Conceptualization (equal); resources (equal); supervision (equal); writing–review and editing (equal). **David Lee Walmsley:** Conceptualization (equal); resources (equal); supervision (equal); writing–review and editing (equal).

## Supporting information

Supplementary Material

## Data Availability

The data that support the findings of this study are available from the corresponding author upon reasonable request.
