# Supplementary material for: Targeting DYRK1A/B kinases to modulate p21‐cyclin D1‐p27 signalling and induce anti‐tumour activity in a model of human glioblastoma
Source: J Cell Mol Med. 2021 Oct 28;25(22):10650–62. doi: 10.1111/jcmm.17002 (PMC8581321; doi:10.1111/jcmm.17002)
Supplement: Supplementary file 1 — Supplementary Material [file JCMM-25-10650-s001.docx]

**Supplemental Information**

**Table S1**. List of antibodies used.

| **Target** | **Supplier** | **Prod. Code** | **Spp.** | **Appl.** | **Dil.** |
| --- | --- | --- | --- | --- | --- |
| pChk1 (S345) | CST | 2348 | R | WB | 1:2500 |
| DYRK1A | CST | 2771 | R | WB | 1:1000 |
| DYRK1B | CST | 5672 | R | WB | 1:1000 |
| Rb2 (p130) | Abcam | ab76234 | R | WB | 1:1000 |
| Rb | CST | 9309 | M | WB | 1:1000 |
| pRb (S807/811) | CST | 9308 | R | WB | 1:1000 |
| pRb (S780) | CST | 9307 | R | WB | 1:1000 |
| Cyclin D1 | CST | 2926 | M | WB | 1:5000 |
|  | Abcam | ab40754 | R | IF | 1:200 |
| p27 | CST | 3686 | R | WB | 1:1000 |
|  |  |  |  | IF | 1:1600 |
| p21 | CST | 2947 | R | WB | 1:2500 |
|  |  |  |  | IF | 1:400 |
| Actin | CST | 4970 | R | WB | 1:10000 |
| pHH3 (S10) | CST | 3377 | R | WB | 1:1000 |
|  |  |  |  | IF | 1:1600 |
| pHH3 (S10) | CST | 9706 | M | IF | 1:400 |
| Ki67 | CST | 9129 | R | IF | 1:400 |
| Cyclin A | CST | 4656 | M | WB | 1:2000 |
| Cyclin B1 | CST | 4135 | M | WB | 1:2500 |
| Cyclin E1 | CST | 4129 | M | WB | 1:2000 |
| pCdc2 (Y15) | CST | 9111 | R | WB | 1:2500 |
| γH2AX | CST | 9718 | R | WB | 1:1000 |
|  |  |  |  | IF | 1:400 |
| mTOR | CST | 2983 | R | WB | 1:1000 |
| pmTOR (S2448) | CST | 2971 | R | WB | 1:1000 |
| p70S6K (T389) | CST | 9205 | R | WB | 1:1000 |
| p4E-BP1 (S65) | CST | 9451 | R | WB | 1:1000 |
| LC3B | CST | 3868 | R | WB | 1:5000 |
| pAKT (S473) | CST | 4060 | R | WB | 1:5000 |

ID., identifier; Appl., application; WB, western blot; IF, immunofluorescence; Spp., species; Dil., dilution; CST, Cell Signaling Technology

**Table S2.** PD marker responses in quiescent U87MG cells following inhibition of DYRK1A/B with VER-239353.

| PD Marker | EC_50_ (nM) | |
| --- | --- | --- |
|  | Monolayer | Spheroid |
| Cyclin D1 | 118 | 132 |
| p21 | 474 | 94 |
| DYRK1B | 282 | 103 |


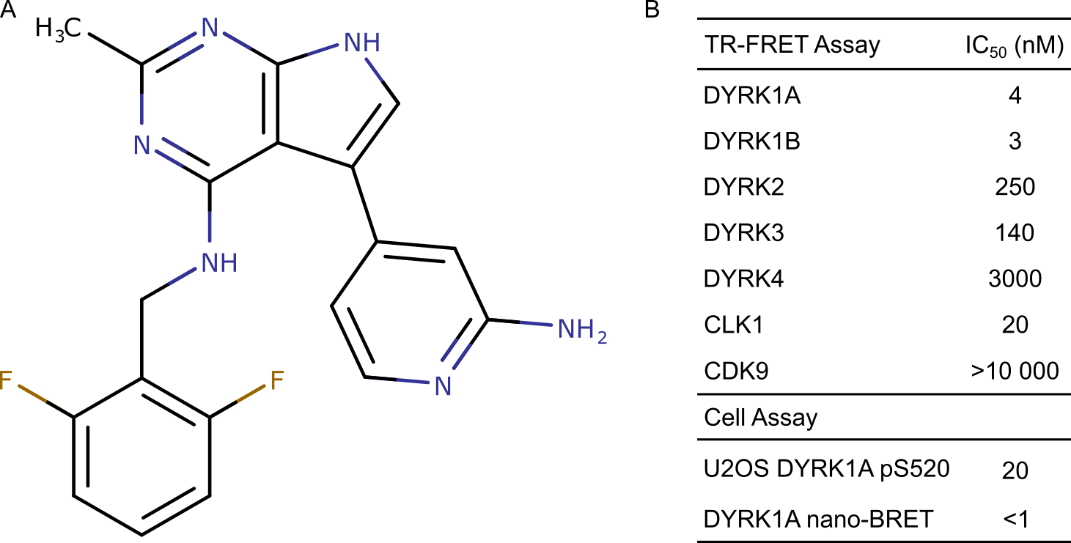


**Figure S1.** Key VER-239353 data.

(A) Chemical structure of VER-239353. (B) *In vitro* kinase and cellular target assay data for VER-239353.


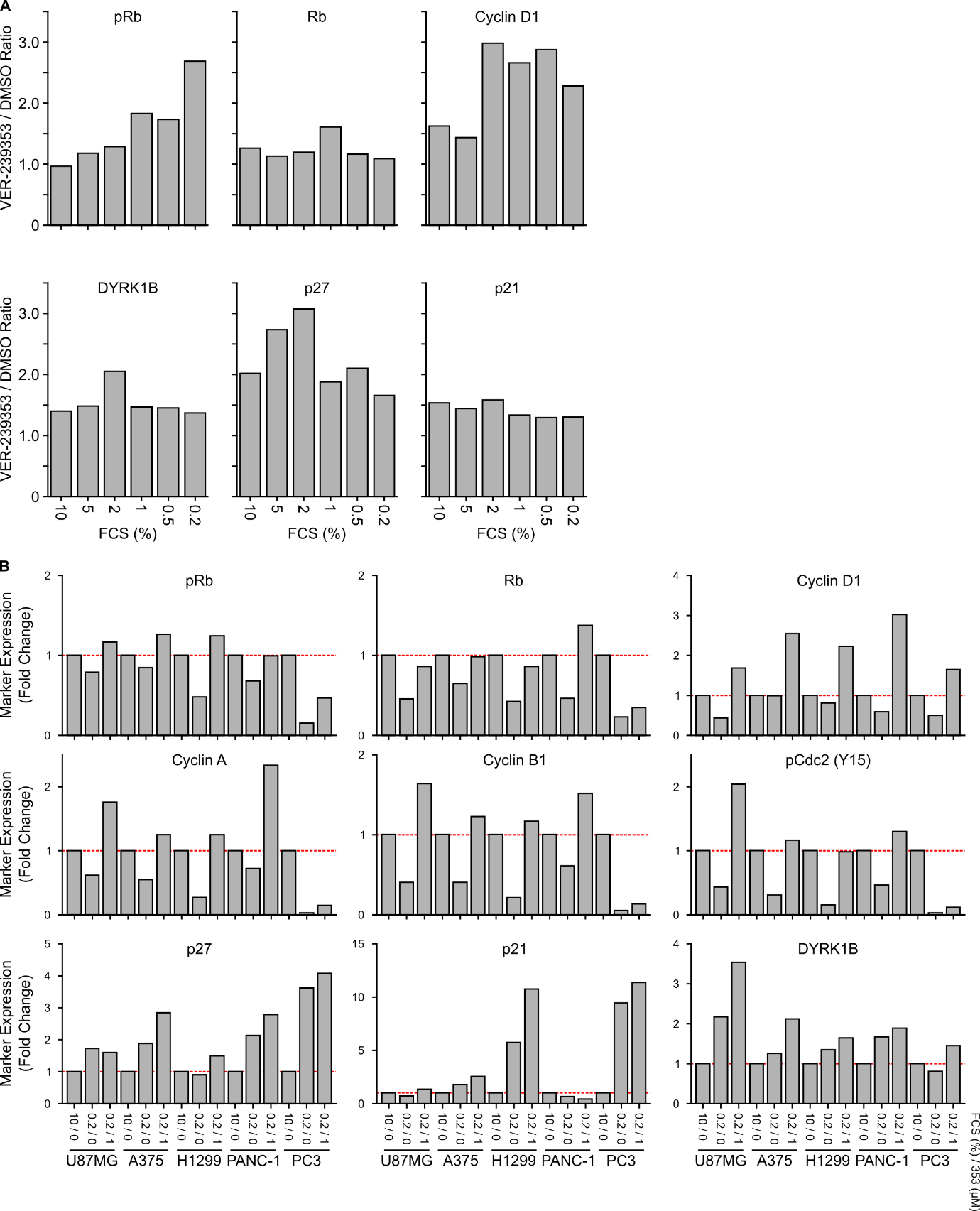


**Figure S2.** Quantification of figures 2A and 2B.

The expression data from Figure 2A (A) or 2B (B) (n=1) was quantified, normalised to Actin and fold change between (A) VER-239353 and DMSO treated or (B) relative to 10% FCS determined.


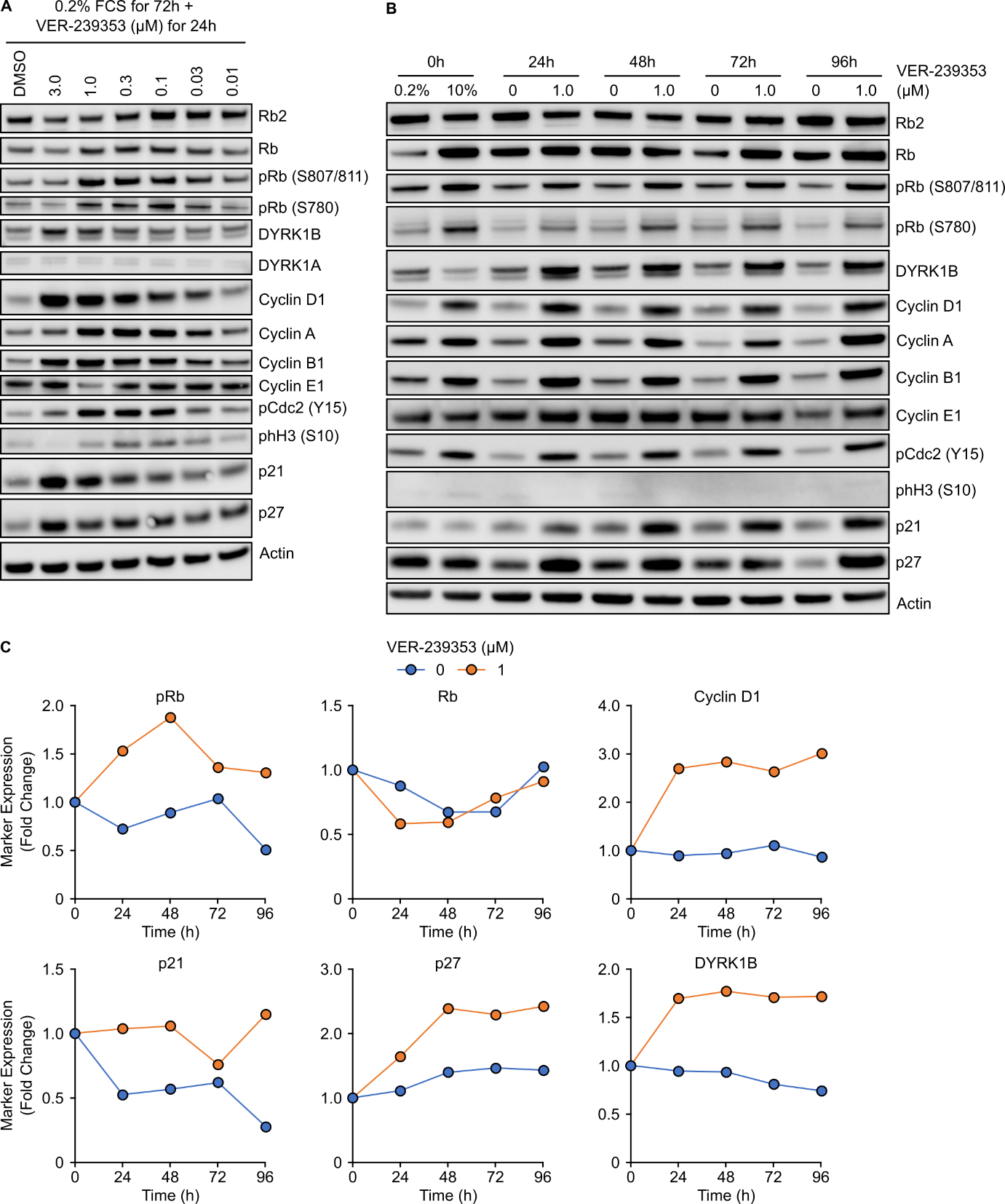


**Figure S3.** Modulation of cell cycle proteins following DYRK1A/B inhibition occurs independently of time.

(A) U87MG cells were cultured in 0.2% FCS for 72 hours before being treated with 0 to 3μM VER-239353 for 24 hours. (B) U87MG cells were cultured in 0.2% FCS for 72 hours before being treated with 0 or 1μM for 24 to 96 hours. Protein marker changes were determined by immunoblotting. (C) The expression data from Figure S3B (n=1) was quantified, normalised to Actin and fold change relative to 0.2%, 0h determined.

**
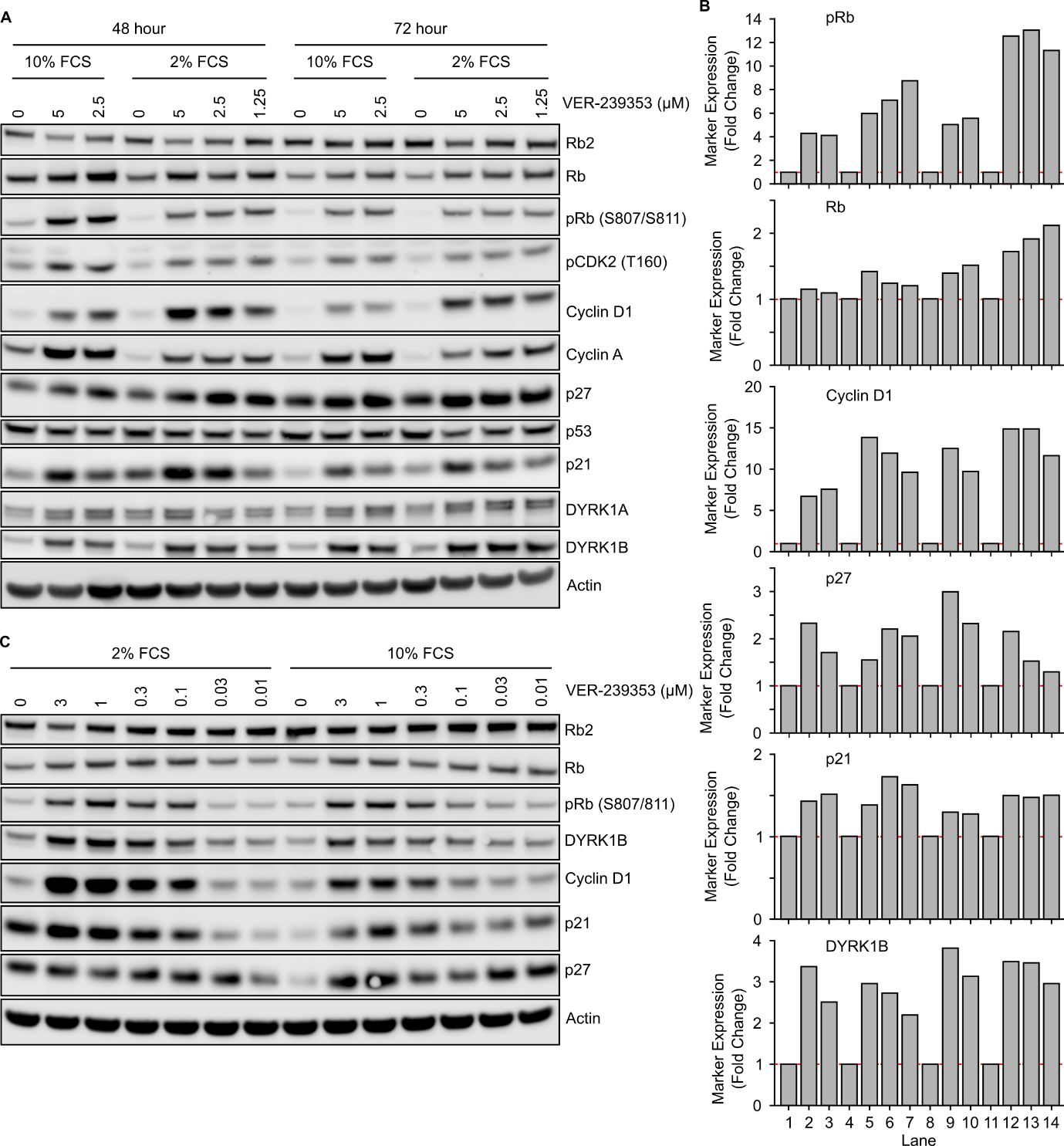
**

**Figure S4.** Modulation of cell cycle proteins following DYRK1A/B inhibition in U87MG multicellular tumour spheroids.

Protein biomarker changes in U87MG multi-cellular tumour spheroids growing in 2% or 10% FCS were determined by immunoblotting following treatment with VER-239353 for (A) 48 or 72 hours, or (C) 48 hours. (B) The expression data from Figure S4A (n=1) was quantified, normalised to Actin and fold change relative to Lane 1 determined.


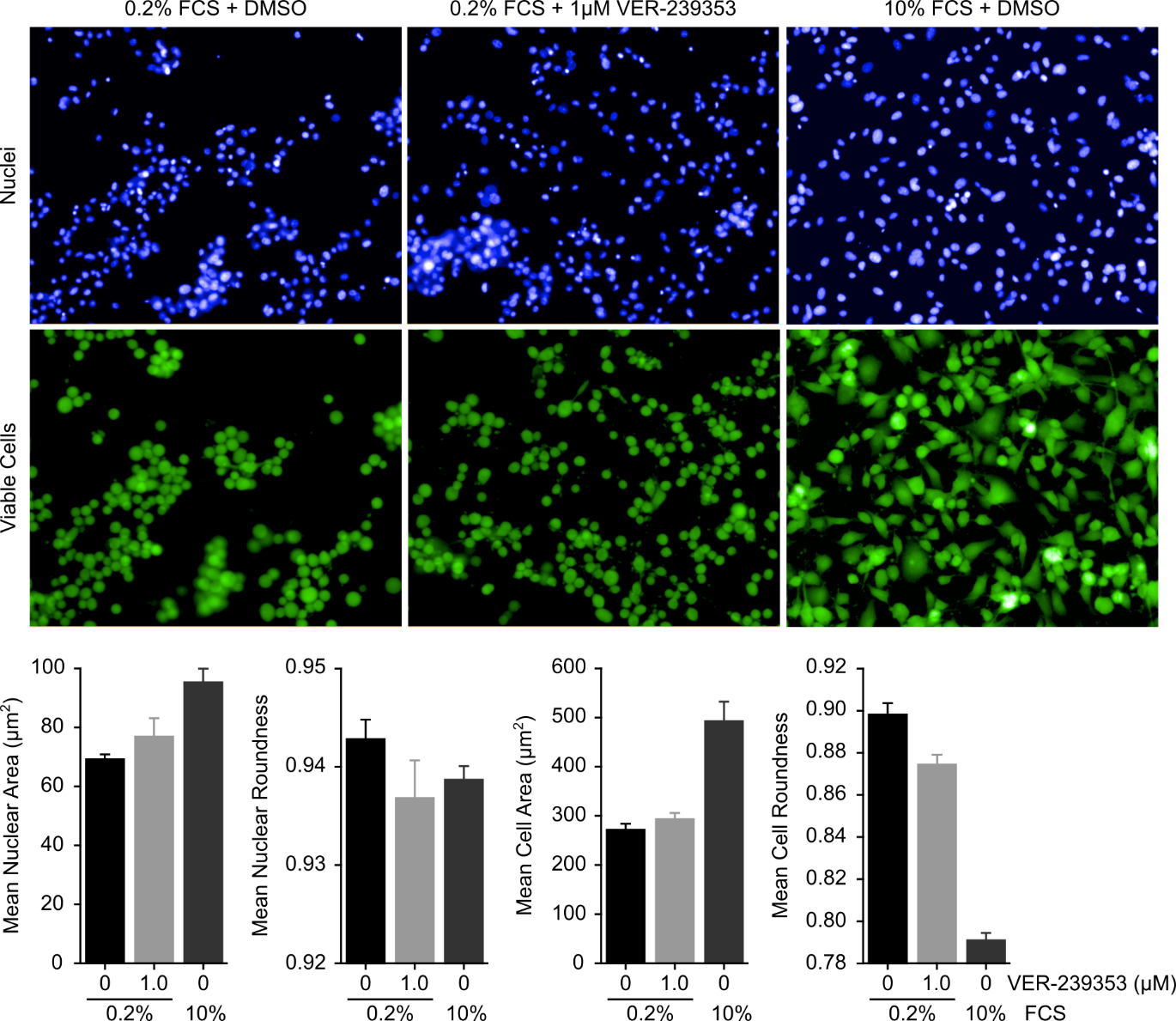


**Figure S5.** Inhibition of DYRK1A/B in quiescent U87MG cells does not affect cell morphology.

U87MG cells were grown in 0.2% or 10% FCS for 72 hours before being treated with 0 or 1μM VER-239353. Live cells were stained with Hoechst 33342 (nuclei) and calcein-AM (viable cells) for 1 hour before being imaged with an Operetta high content imager. Cell and nuclear parameters were determined using Harmony software. Values are the mean of 8 determinations ± SD.


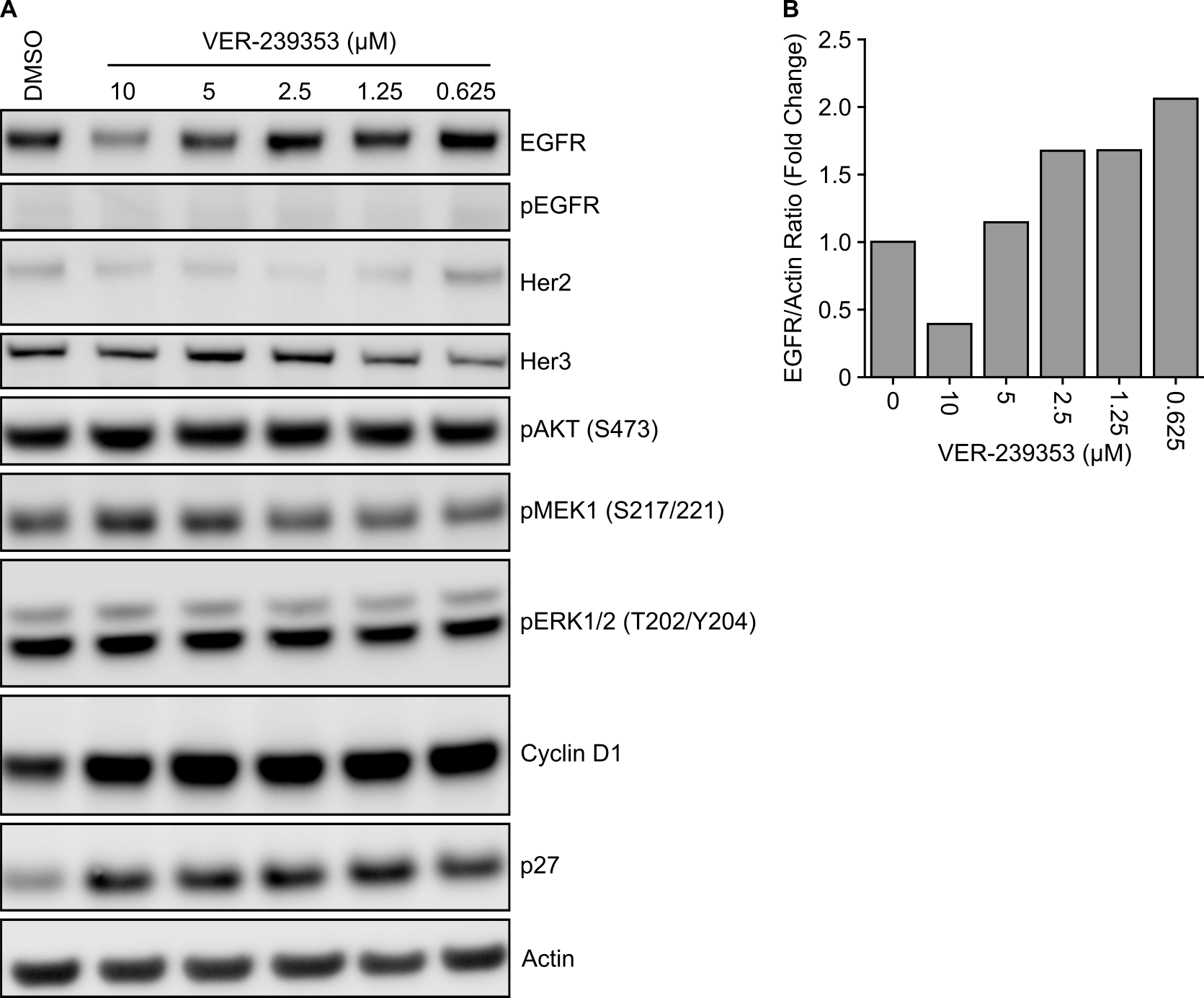


**Figure S6.** Inhibition of DYRK1A/B in U87MG cells does not downregulate EGFR.

(A) U87MG cells growing in 10% FCS were treated with the indicated concentrations of VER-239353 for 24 hours and protein marker changes determined by western blotting. The experiment was conducted 3 times and a representative set of blots is shown. (B) The EGFR expression data from (A) was quantified (n=1), normalised to Actin and fold change relative to DMSO control determined.


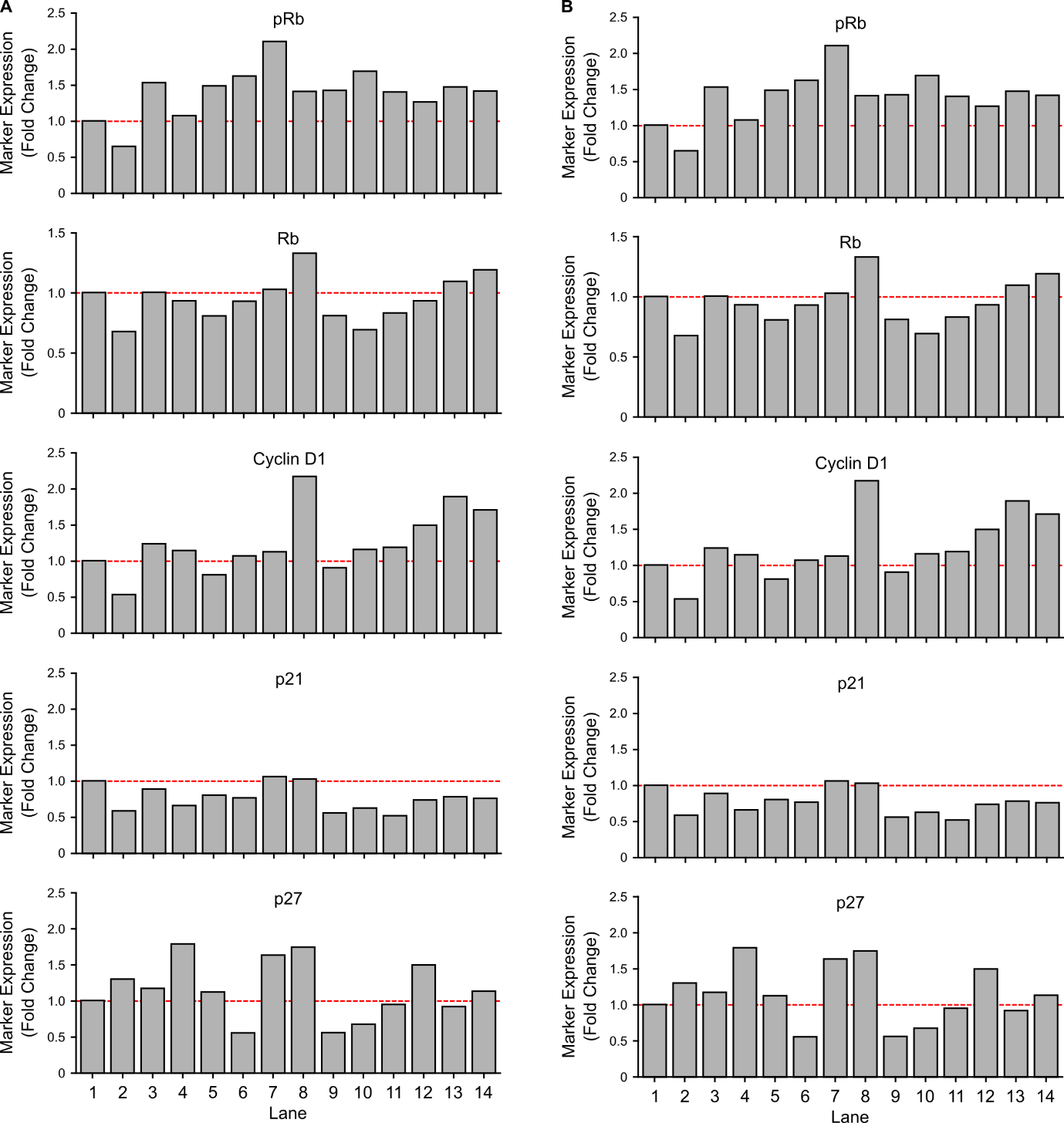


**Figure S7.** Quantification of Figures 5C & 5D

The expression data from Figure 5C (A) or 5D (B) was quantified, normalised to Actin and fold change relative to Lane 1 determined.
